# A new approach to design safe CNTs with an understanding of redox potential

**DOI:** 10.1186/1743-8977-10-44

**Published:** 2013-09-02

**Authors:** Shuji Tsuruoka, Flemming R Cassee, Vincent Castranova

**Affiliations:** 1 Research Center for Exotic Nanocarbons, Shinshu Univerity, 4-17-1 Wakasato, Nagano 380-8553, Japan; 2 National Institute for Public Health & Environment, Ministry of Health, Welfare & Sport, A.van Leeuwenhoeklaan, 9 3721, Bilthoven, MA, The Netherlands; 3 Institute for Risk Assessment Sciences, Utrecht University, Utrecht, the Netherlands; 4 National Institute for Occupational Safety and Heatlth, Pathology & Physiology Research Branch, 1095 Willowdale Rd. (M/S2015), 26505-2888, Morgantown, WV, USA

## Abstract

**Background:**

Carbon nanotubes (CNTs) are being increasingly industrialized and applied for various products. As of today, although several toxicological evaluations of CNTs have been conducted, designing safer CNTs is not practiced because reaction kinetics of CNTs with bioactive species is not fully understood.

**Results:**

The authors propose a kinetic mechanism to establish designing safe CNTs as a new goal. According to a literature search on the behavior of CNTs and the effects of impurities, it is found that chemical reactions on CNT surface are attributed to redox reactions involving metal impurities and carbon structures at the CNT surface.

**Conclusion:**

A new goal is proposed to design safer CNTs using the redox potential hypothesis. The value of this hypothesis must be practically investigated and proven through the further experiments.

## Background

Designing safe Carbon Nanotubes (CNTs) is an important goal but requires elucidation of mechanisms that drive biological responses to CNTs. Importance of physicochemical properties is often suggested in articles, but the relative importance of specific properties has not been defined explicitly. Two critical points concerning CNT safety evaluations are: bioactivity of CNTs and impurities, and the fiber paradigm. The latter not only applies to CNTs but also other nanowires and micro fibers. Thus, we would like to discuss the former, bioactivity, that is, chemical reactions on the CNT surface. Recent investigations suggest that there exists an intrinsic mechanism of redox potential with CNTs and its metal impurities [1-5]. It must be noted that redox means both oxidation and reduction.

## Results

Redox reactions of CNTs are attributed to metal impurities and the structure of carbon lattice. First, metal impurities are brought by synthesizing catalysts that are transition metals, such as Fe, Ni, Co. Mo, and so on. Among them, iron will be discussed as a typical model. A good method to remove metal impurities from CNTs is a wash in concentrated nitric acid at 350°C [6]. A strong acid wash may alter the surface of carbon nanotubes, modifying surface reactivity. Thus, it is best to avoid strong acid washing that would damage CNTs. Interestingly, Guo et al. [1] reported that Fe_2_O_3_ and FeC encapsulated by carbons cannot be mobilized nor be bioavailable to an acid wash. Furthermore, Liu et al. [7] discussed that it was not necessary to remove metal impurities from CNTs as long as those metals were enclosed and not bioavailable. Thus, it is possible to manage iron impurities by an appropriate acid wash, and bioactivity will be reduced as long as remaining impurities are not bioavailable.

To various degrees, transition metal impurities are usually oxidative to peroxides while metal oxides are relatively stable. It is known that Fe (II) or Fe^2+^ ion generates OH radicals (OH•), a form of reactive oxygen species (ROS), by the Fenton reaction, and that ROS induce inflammation of tissues. As Fe^3+^ generated by the oxidation is again reduced to Fe^2+^ with peroxide, iron can continue to cause oxidant-induced inflammation in living tissues. In contrast, Fe (III) oxide (Fe_2_O_3_) and carbide (FeC) do not generate ROS, because Fe (III) cannot be an electron donor except upon treatment with a strong reduction agent. Fe (II) is supplied not only externally as metal impurities but also internally in a living body. Since Fe (II) essentially catalyzes peroxide generating OH•, redox (reduction) reactions are required to eliminate the radicals. A question is what redox potential do CNTs exhibit.

Redox potential determines the reaction tendency of chemicals in a system. CNTs inevitably have dangling bonds at which unpaired electrons can be easily exchanged with the other species. If those dangling bonds donate electrons to OH•, CNTs become ROS quenchers. According to recent findings [8-11], CNTs scavenge ROS so that those dangling bonds work as electron donors. In other words, CNTs can potentially reduce OH•. If this redox potential hypothesis is true for CNTs, they may decrease oxidant-induced inflammation of tissues. One should be able to stoichiometrically predict oxidant stress once redox potential of CNTs in a reaction system is determined. This hypothesis might apply for the other nanomaterials as well. Furthermore, a systematic protocol based on chemical reaction kinetics can be applied to develop predictive *in vitro* assays of redox potential for various types of CNTs. Accumulating those redox reaction data, guidance to design safer CNTs can be developed. In conclusion, redox potential might be a useful tool to estimate ROS generation and bioactivity with CNTs. To utilize it, we have to investigate redox potentials of biological systems further and identify the role of oxidant stress in the toxicity of CNTs.

## Conclusion

It is hypothesized that radical generation and degeneration (quenching) can be determined by redox potential in a reaction system with CNTs and impurities. A goal to design safer CNTs is proposed based on the redox potential. It must be practically investigated and proven through the further experiments.

## Abbreviations

CNTs: Carbon nanotubes; ROS: Reactive oxygen species.

## Authors’ contributions

All authors equally participated to conduct a literature search and laboratory studies and to draft this text. All authors read and approved the final manuscript.
